# Monitoring Colorectal Cancer Screening at Scale: Conformance Checking and Bottleneck Detection in Northern Portugal

**DOI:** 10.1177/21501319261458040

**Published:** 2026-06-11

**Authors:** Hugo Monteiro, Bárbara Gonçalves, Ricardo Martinho, Óscar Felgueiras, Carlos Martins

**Affiliations:** 1 MEDCIDS - Departamento de Medicina da Comunidade, Informação e Decisão em Saúde, Faculdade de Medicina da Universidade do Porto, Portugal; 2 131674 Department of Mathematics, Faculdade de Ciências da Universidade do Porto, Portugal; 3 RISE Health, University of Leiria and Oeste, Portugal; 4 Centre for Health Technology and Services Research (CINTESIS@RISE), 26706 University of Porto, Portugal

**Keywords:** colorectal cancer screening, primary care, follow-up, process evaluation, process mining

## Abstract

**Objective:**

To evaluate follow-up after positive faecal immunochemical test (FIT) results in a population-based colorectal cancer screening programme and identify pathway drift and operational bottlenecks relevant to primary care coordination.

**Methods:**

We conducted a retrospective descriptive service evaluation using time-stamped administrative event-log data from the Northern Region of Portugal. The full dataset covered 2018 to 2025 and included 6.96 million events from 1.18 million invited individuals. The main comparative analysis used incident screening episodes starting in 2022-2023 (n = 320 330) and 2024-2025 (n = 135 213), each followed for 365 days from invitation mailing. Key variables were pathway variants, conformance precision, alignment cost, and dwell time between operational steps from invitation to colonoscopy-related activity.

**Results:**

Three dominant variants accounted for more than 95% of episodes in both cohorts. Precision for the 2024-2025 cohort was 0.65 against its own model but fell to 0.45 when assessed against the 2022-2023 baseline model, indicating behavioural drift despite fitness remaining close to 1.00. Median alignment cost remained low overall (7-11 normalised units), but p90 values of 11-16 showed that deviations were concentrated in subsets of cases. Among FIT-positive episodes entering primary care follow-up, approximately 60% proceeded to colonoscopy within 365 days. Dwell time for the FIT mailed to FIT returned transition increased by 31% in the POST cohort, and laboratory result-to-follow-up transitions also lengthened.

**Conclusions:**

Routine pathway monitoring identified incomplete follow-up after positive FIT results and operational drift not captured by conventional participation indicators alone. These findings support targeted action in primary care and programme management, particularly reminder systems, referral tracking, and protected capacity for timely diagnostic colonoscopy.

## Introduction

### The Colorectal Cancer Screening Program

Colorectal cancer (CRC) remains a leading cause of morbidity and mortality in Europe. Population-based screening programs that detect precancerous lesions and early-stage tumours can markedly reduce both incidence and death rates when uptake is high.^1^ European guidelines recommend participation rates between 45 % and 65 % for stool-based tests and completion of colonoscopy in more than 90 % of individuals with a positive result, with timely scheduling recommended.^2^ Yet adherence varies widely across countries: participation rates vary substantially, and completion of diagnostic colonoscopy after a positive faecal immunochemical test (FIT) rarely reaches the desired threshold. Barriers to uptake include socioeconomic inequities, insufficient patient education, fear of invasive procedures and a fatalistic perception of cancer.^2-4^ These realities underscore the need to audit how individuals traverse the screening pathway and to design interventions that improve engagement. Even in organised population-based programs, execution varies with resources, operations and shocks such as COVID-19, which directly affect performance.^5-8^

Portugal’s national program targets adults aged 50-74 years and offers biennial FIT followed by colonoscopy for individuals with positive results. The program has gradually expanded from pilots to full national coverage, but by 2022 only about one-third of the eligible population had been invited, and the observed participation rate was 41 %.^9^ Uptake varies across regions, with the north exceeding 50 % and the Alentejo region falling below 25 %. The national strategy aims to invite 95 % of the target population and achieve 65 % participation by 2030.^9^ To address organisational challenges, the Ministry of Health established a National Coordination Centre for Population-Based Screening Programs in 2023.^9^ This unit oversees CRC, breast and cervical screening, harmonises protocols and monitors performance across local health units. Early evaluations of the northern region reported participation rates of 29 % of invites that returned the FIT kit and 60 % of positive cases proceeded to colonoscopy. The analysis identified repeated invitation loops, delays in returning kits and limited follow-up capacity as key bottlenecks.^10^

Evidence from Galicia’s Rapid Referral Pathway showed a median time to colonoscopy of 7 days versus 34 days in usual care, illustrating the impact of targeted pathway design.^11^ Therefore, consistent process metrics and transparent governance are essential to reach the EU objective, providing guidance on improving patient pathways from invitation to colonoscopy.^12^

### Digital Transformation and Process Analytics in Health

Digital technology including cloud computing and artificial intelligence is reshaping healthcare delivery.^13^ Process mining is emerging as a novel management support tool in healthcare, offering innovative ways to analyse and optimise complex systems. This approach can be applied in multiple settings, where it uncovers temporal patterns from patient-reported outcome data and enhances traditional machine learning analyses by providing process-level insights.^14^

Process mining sits at the intersection of data science and process management. It extracts knowledge from events to discover how processes unfold, check conformity with a reference model and enhance processes through targeted modifications. By bridging traditional process analysis with data-driven techniques such as machine learning, process mining can transform raw logs into actionable models. A review of process mining in healthcare showed that process discovery algorithms are particularly relevant due to their ability to handle noise and complexity.^15^ Process mining has proved effective in analysing clinical workflows, clarifying guidelines and supporting decision-making; however, it requires attention to data quality and intuitive visualisation. Recent cost-mining studies further highlight that process mining can quantify costs along patient pathways in multiple settings.^16^

Clinical guideline compliance can also be assessed with process mining. In a study of rectal cancer treatment, researchers translated the European Society for Medical Oncology guidelines into a Pseudo-Workflow Formalism and implemented the model in the pMineR software.^17^ Applying conformance checking to a real-world cohort, the study identified patient subgroups deviating from recommended sequences and highlighted specific treatment choices (e.g. increased radiotherapy dose) as sources of deviation. This demonstrates how process mining can detect adherence gaps and resource constraints affect care delivery.

Building on previous analyses of performance and resilience in the same population-based colorectal cancer screening program, this study evaluates follow-up after positive FIT results, with a specific focus on conformance and behavioural drift across program periods. Using inductive and guideline-driven models combined with alignment-based and token-replay metrics, we quantify how real-world execution deviates from both the observed as-is baseline and an idealised guideline model. This provides a structured view of where pathway deviations concentrate, how they evolved after program re-organisation, and how they may inform capacity planning and quality improvement.

## Methods

We analysed the regional CRC screening event log, covering 1 January 2018 to 1 August 2025. Each record contained a case identifier, the activity name (invitation mailing, FIT kit mailing, FIT kit returns, laboratory return, laboratory result, primary care forwarding, primary care observation, colonoscopy centre), a timestamp and contextual attributes. The raw dataset includes approximately 6.96 million events across 1.18 million distinct cases within the North Region.

New screening episodes were defined by the first recorded event (index date = invitation mailing). Two incident cohorts were analysed: cases from 1 January 2022 to 31 December 2023, formed the pre-2024 cohort (PRE); cases with an index date ≥ 1 January 2024 formed the post-2024 cohort (POST). For each case we retained events occurring within 365 days after the index; later events were excluded. This horizon covers a typical CRC screening cycle and supports comparability across start times. Given the log scale, we performed case-level sampling only where specified for computationally intensive alignments, preserving full traces for fitness and precision (the extent to which the model permits only observed behaviour) assessments.

All data handling, normalisation and process mining were executed in Python 3.10 using the PM4Py 2.2.19 library.^18^ Additional visual analytics were conducted in R 4.3 using the bupaR and edeaR packages.

Preparing the log for process mining involved several iterative steps. The raw event stream was cleaned, harmonised, and standardised to preserve clinical meaning while ensuring process-analytic consistency. Timestamps were converted to a common format, duplicated events were removed, and activity labels were aligned across data sources before cohort extraction.

We applied two complementary discovery algorithms. Heuristics Miner was used to obtain an initial, highly readable view of dominant behaviour, while Inductive Miner was used for the primary analytical models because it produces sound Petri nets and supports robust conformance checking.

All conformance results presented in this study rely exclusively on the Inductive Miner models and on the guideline-based reference model; outputs from Heuristics Miner were used only during exploratory analysis and are not reported quantitatively.

We generated visuals and computed model complexity metrics (transitions, places, arcs, loops, duplicate labels). Special attention was given to the re-invitation loop (invitation mailing → PCC_FIT_rejection → invitation mailing) and to the identification of new transitions introduced in 2024-2025 (e.g. primary care observation after colonoscopy, which were expected post incomplete colonoscopies, mainly due to bad preparation).

Observed processes were compared with two reference models: (1) an idealised guideline-based model was derived from national and international CRC screening norms (Invitation → FIT mailing → FIT return → Laboratory analysis → [Primary Care Consultation → Colonoscopy for FIT-positive cases]).^2^ We formalised this sequence and converted it into a Petri net. This model provides a strict upper bound for precision. (2) the data-driven baseline Inductive Miner model discovered from the PRE cohort, which captured prevalent behaviours and served as a realistic baseline for cross-period evaluation. Models were iteratively reviewed in collaboration with domain experts.

Conformance was quantified using three metrics. Token replay fitness measures how well the model reproduces actual sequences of events. Values near 1 indicate high replayability. Alignment-based fitness (cost) quantifies how much deviation exists between observed behaviour and the model, using an algorithm that assigns penalties for missing or extra steps. We report both median and 90th percentile values to capture typical and outlier cases. Precision (ETConformance) applies penalties for unused model behaviour enabled during replay; higher values indicate less permissive models.

Deviations were classified as insertions, omissions or swaps and linked to operational causes, such as re-invitation loops, kit-return delays, and limited follow-up capacity. Bottlenecks were profiled with mean, p90 and trimmed mean (5-95 %) dwell times; zeros were masked and transitions ranked by p90. Analyses were stratified by cohort and calendar year to contextualise observed changes. This framework allows clinicians to monitor workflows at high level and drill down on specific transitions when deviations emerge, thereby increasing intervention efficiency.

**Figure 1.**
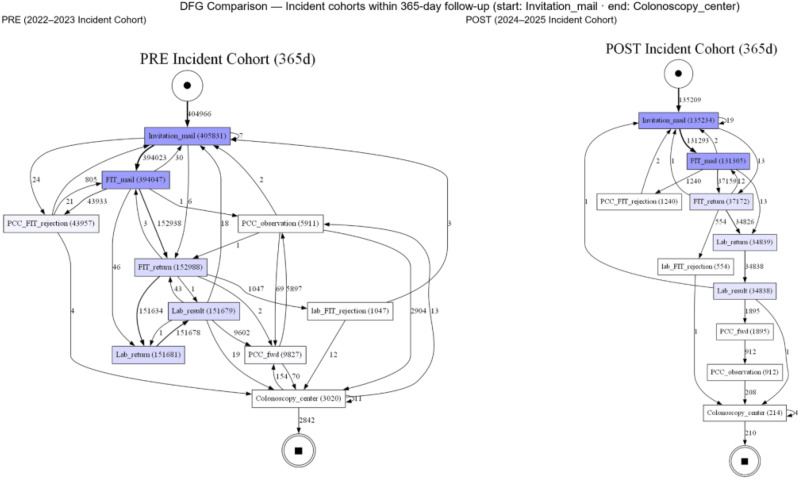
Directly-follows graph comparing PRE (2022-2023) and POST (2024-2025) cohorts within a 365-day follow-up anchored at invitation mailing as first event. The dominant re-invitation loop and the high-frequency cascade from invitation through FIT completion to laboratory processing are evident in both periods (darker blue nodes)

The PRE cohort (2022-2023) included 320 330 cases and 1 027 502 events, and the POST cohort (2024-2025) included 135 213 cases and 378 209 events (Figure 1). Both cohorts share the same core activities. We performed discovery with Heuristics (HM) and Inductive Miner (IM) on both logs; the IM (PRE) model served as the empirical baseline for cross-period evaluation.

Access to the event log was authorized through the institutional responsibilities of the screening coordination structure that holds the data, and the analysis was conducted on de-identified administrative records under ethics approval CE/2023/96. Additional technical details and supplementary analyses are provided in the Supplemental Material.

## Results

### Cohorts and Dominant Pathways

**Figure 2.**
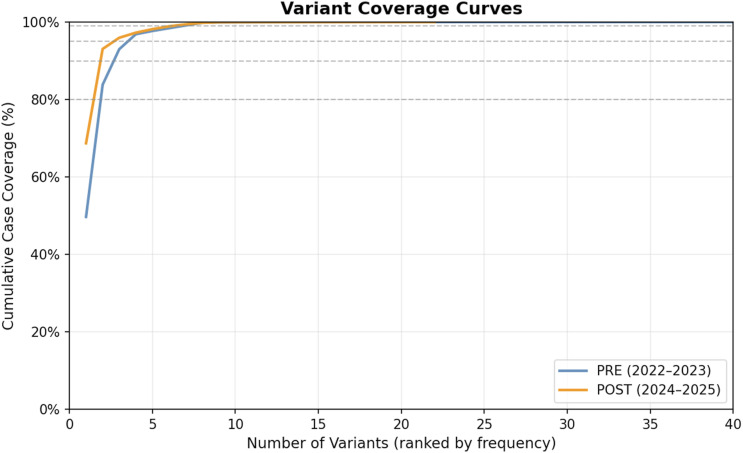
Variant coverage curves. Both PRE and POST reach 95% case coverage with three variants

The PRE cohort comprised 320 330 episodes and 1 027 502 events, while the POST cohort comprised 135 213 episodes and 378 209 events (Figure 1). In both periods, pathway behaviour was highly concentrated: three dominant variants captured more than 95% of episodes (Figure 2).

### Behavioural Drift Across Programme Periods

**Figure 3.**
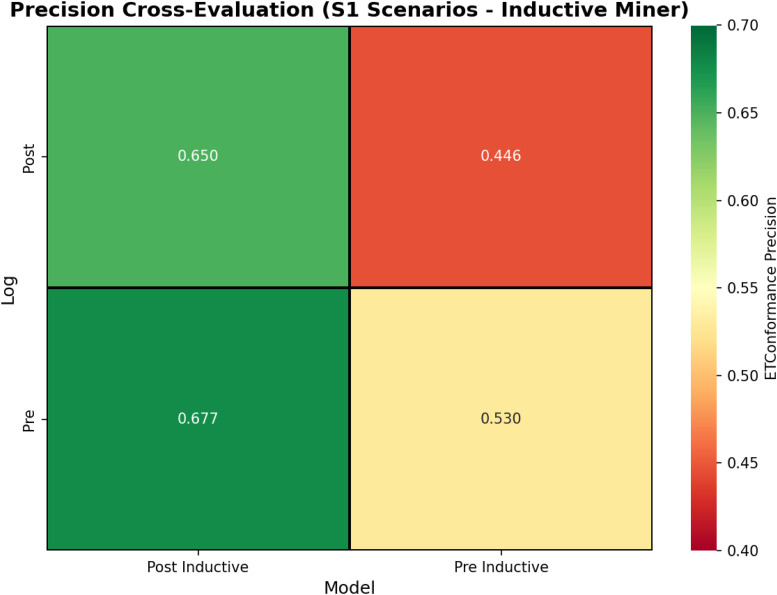
ETConformance precision heatmap. Cross-evaluation (POST-on-PRE) shows precision dropping to 0.45, revealing behavioural drift

Fitness remained close to 1.00 in all Inductive Miner scenarios and did not distinguish meaningfully between periods. Precision provided the clearest signal of behavioural drift: it was 0.53 for PRE and 0.65 for POST in self-evaluation, but fell to 0.45 when POST traces were replayed on the PRE model (Table 1 and Figure 3).

**Table 1.** Conformance Metrics Summary

Scenario	Log	Model	Miner	Token fitness	Precision_	Median
Self	PRE	PRE	Inductive	1.0000	0.530	8.88
Self	POST	POST	Inductive	1.0000	0.650	11.01
Cross	POST	PRE	Inductive	1.0000	0.446	8.04
Cross	PRE	POST	Inductive	1.0000	0.677	13.80

**Figure 4.**
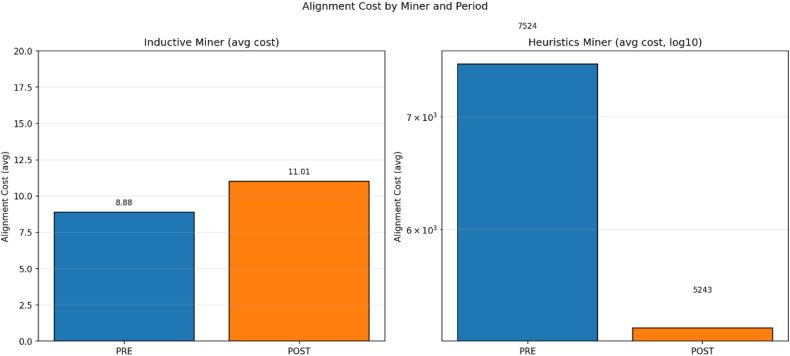
Alignment cost distribution by cohort and miner. Median values remained low, but higher p90 values indicated that deviations were concentrated in subsets of cases; Heuristics miners showed broader variance than Inductive miners

Alignment cost remained low on average, with median values between 7 and 11 normalised units, but p90 values between 11 and 16 showed that deviations were concentrated in subsets of cases rather than distributed evenly across the programme (Figure 4).

### Follow-Up After Positive FIT and Bottlenecks

**Figure 5.**
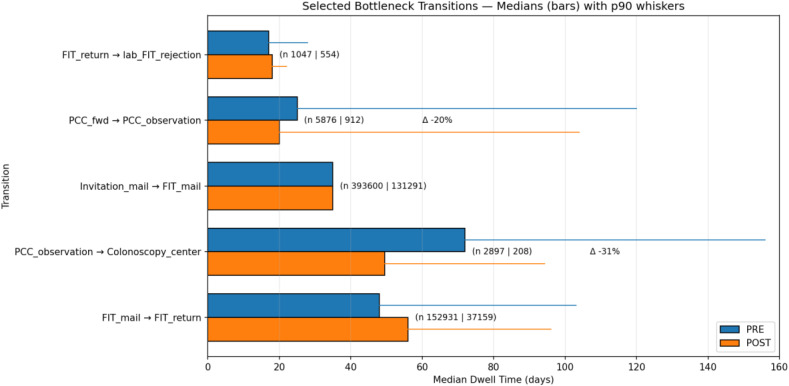
Selected bottleneck transitions - comparison between PRE and POST. Bars show median dwell times (days) with p90 whiskers; the FIT mailed to FIT returned transition increased by 31% in the POST cohort, whereas primary care observation to colonoscopy centre decreased by about 31%, reflecting divergent dynamics across the pathway

The most relevant service-level finding concerned follow-up after positive FIT results. Among FIT-positive episodes entering primary care follow-up, approximately 60% proceeded to colonoscopy within 365 days. This suggests that the main challenge is not only participation in initial screening but also completion of the diagnostic pathway after a positive result. Dwell-time analysis showed that the FIT mailed to FIT returned transition increased by 31% in the POST cohort (Figure 5). Laboratory result-to-follow-up transitions also lengthened, indicating pressure points after test processing and before definitive investigation. By contrast, some downstream transitions shortened among those who had already reached later stages of the workflow.

## Discussion

### Follow-Up After Positive FIT (Closing the Loop)

Within the 365-day window, we observed incomplete follow-up of positive FIT results (approximately 60% colonoscopy completion). Fast-track models show that protected capacity and streamlined referral can cut waits significantly (e.g. reducing median time from ∼34 days to ∼7 days).^11^ Similar approaches, including templated referrals, pooled lists and protected FIT-positive slots, may help shorten these hand-offs and improve completion.

To avoid over-permissive discovery, we used the guideline-based reference model as the normative “should-be” comparator, while treating Heuristics Miner outputs as exploratory visual checks only (not used for quantitative conformance reporting). In practice, the as-is model achieved near-perfect replay of cases with very little correction needed but was more permissive, while the strict model allowed almost only guideline-like behaviour yet missed part of real-world variability and required more corrections. Looking at both prevents false reassurance from high fitness alone (over-permissive models) or from high precision alone (models that exclude legitimate variation). To make the signals clinically useful, we focused on precision to detect period drift when fitness saturates, and on deviation-cost summaries (median/p90) to localise where individual cases depart from the model; reporting both raw deviations and normalised costs improves interpretability for quality-improvement work. Our combined approach offers a practical, reproducible template for guideline-driven services and aligns with recent reviews highlighting the need for clinically interpretable and standardised conformance measurement in healthcare process mining.^19-21^

Our analysis relies on administrative event logs, which may contain misclassified or missing records; thus, some deviations may reflect data artefacts rather than true process failures. The 365-day follow-up window censors very late completions, outside observation, and may affect loop frequency and deviation costs. The POST-2024 cohort has a shorter observation window than PRE-2024 cohort, introducing a horizon bias that likely under-captures re-invitation loops and can inflate apparent conformance. Finally, our estimates reflect the Northern Region’s demographic and organisational context; while issues such as participation gaps and follow-up delays are common in population screening, absolute metrics may differ elsewhere.

Conformance indicators are sensitive to model structure and parameter choices. We mitigated this by comparing multiple models and metrics but recommend expert review of high-cost cases to distinguish benign variation from true non-compliance.

Finally, while we identified correlations between process deviations and potential causes (e.g. repeated invites suggesting low engagement or missing follow-up suggesting resource issues), we cannot prove causality from this observational data alone. External factors (such as individual health-seeking behaviour or simultaneous participation in other screenings) could influence these patterns. Our recommendations (like adding reminders or fast-tracking colonoscopies) are based on logical inference and literature support, but their actual impact should be evaluated, ideally through prospective studies or pilot interventions.

Future work could build on this work in several ways, expanding process mining techniques applied to healthcare.^22^ One important next step is to test targeted interventions informed by our findings. For example, introducing a pre-invitation phone call for those who ignored an initial mail, or establishing a dedicated navigation service for FIT-positive patients to ensure they complete colonoscopy. By re-running the conformance analysis after such changes, one could directly measure improvements (e.g. a reduction in the re-invitation loop frequency or higher precision and lower alignment-cost p90 against the ideal model). Additionally, exploring other process-mining techniques such as variant analysis or machine-learning clustering on the event log may uncover if certain demographic groups or local health units have systematically different process flows.^23^ This could help tailor interventions more precisely. There is also opportunity to extend conformance checking to other screening programs (such as breast or cervical cancer screening), as the National Coordination Centre oversees multiple programs. Comparing conformance across programs might identify common system-wide bottlenecks or successful strategies that could be applied across the different programs. Lastly, incorporating clinical outcomes (e.g. cancer detection rates, stage at diagnosis) with the process data would allow us to assess not just process adherence but its ultimate impact on patient outcomes, closing the loop between operational efficiency and public health benefit. Recent frameworks for health-equity monitoring and continuous improvement using Kaizen principles offer promising templates for integrating process mining into broader quality-improvement and equity-assessment efforts.^14,22^

## Conclusion

In a mature, high-volume screening programme, fitness alone did not discriminate between periods; only precision and alignment cost revealed behavioural drift and actionable deviations. Cross-period evaluation showed a marked precision decline (0.65 to 0.45) despite fitness remaining at 1.00, and only approximately 60% of FIT-positive episodes reached colonoscopy within 365 days, with increased dwell time for the FIT mailed to FIT returned transition. By combining a guideline-based reference model with cohort-specific discovered models, this reproducible workflow analysis enables routine, pathway-level monitoring. However, identifying bottlenecks is only the first step. To bridge the gap between a positive FIT result and a diagnostic colonoscopy, health systems must implement active triage models - such as templated fast-track referrals, explicit prioritisation criteria, and protected endoscopy slots.^24^ Reducing post-laboratory turnaround times eliminates critical diagnostic delays and aligns with emerging literature on cost mining and continuous quality-improvement frameworks.^16,22^ More broadly, recent perspective work suggests that process mining can also complement other data-driven approaches in longitudinal digital-health datasets.^25^

## Supplemental Material

Supplemental Material - Monitoring Colorectal Cancer Screening at Scale: Conformance Checking and Bottleneck Detection in Northern Portugal Supplemental material for Monitoring Colorectal Cancer Screening at Scale: Conformance Checking and Bottleneck Detection in Northern Portugal by Hugo Monteiro, Barbara Gonçalves, Ricardo Martinho, Oscar Felgueiras and Carlos Martins in Journal of Primary Care & Community Health.

## Data Availability

The administrative event log contains information held by a national coordination entity of the Ministry of Health and cannot be publicly released. Aggregated data and analytical code are available from the corresponding author on reasonable request.*
