# Supplementary material for: Monitoring Colorectal Cancer Screening at Scale: Conformance Checking and Bottleneck Detection in Northern Portugal
Source: J Prim Care Community Health. 2026 Jun 11;17:21501319261458040. doi: 10.1177/21501319261458040 (PMC13261011; doi:10.1177/21501319261458040)
Supplement: Supplemental Material - Monitoring Colorectal Cancer Screening at Scale: Conformance Checking and Bottleneck Detection in Northern Portugal [file sj-pdf-1-jpc-10.1177_21501319261458040.pdf]

Supplementary Material

S1. Cycle Analysis

The screening program is organized as annual (365-day) cycles. Multi-cycle participation indicates repeated engagement across invitations; the figures below summarize cycle counts and participation intensity.

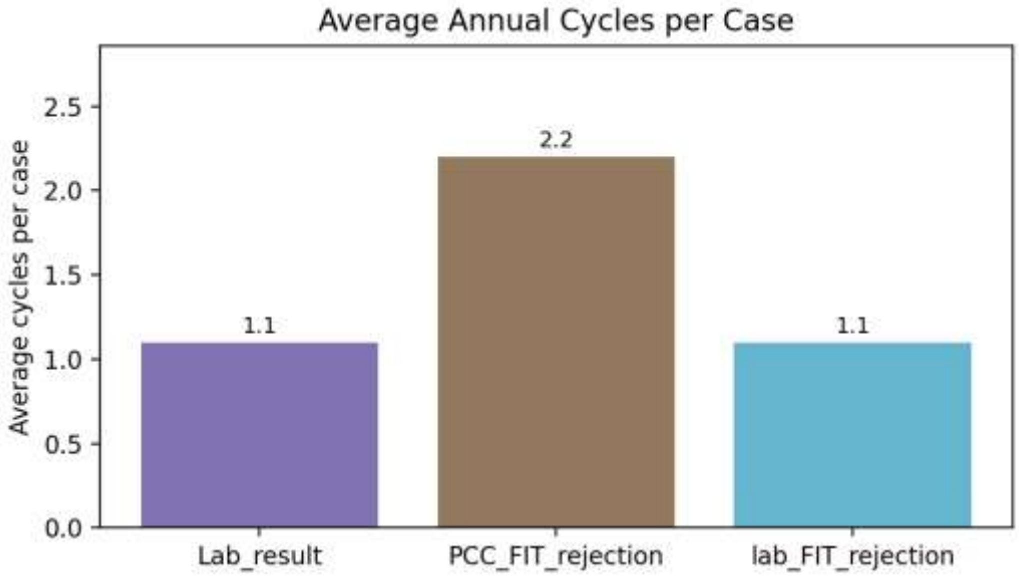

Figure S1. Average cycles per case. Most participants complete one or two cycles within the observation horizon, and a small subset engages in three or more cycles, highlighting sustained adherence.

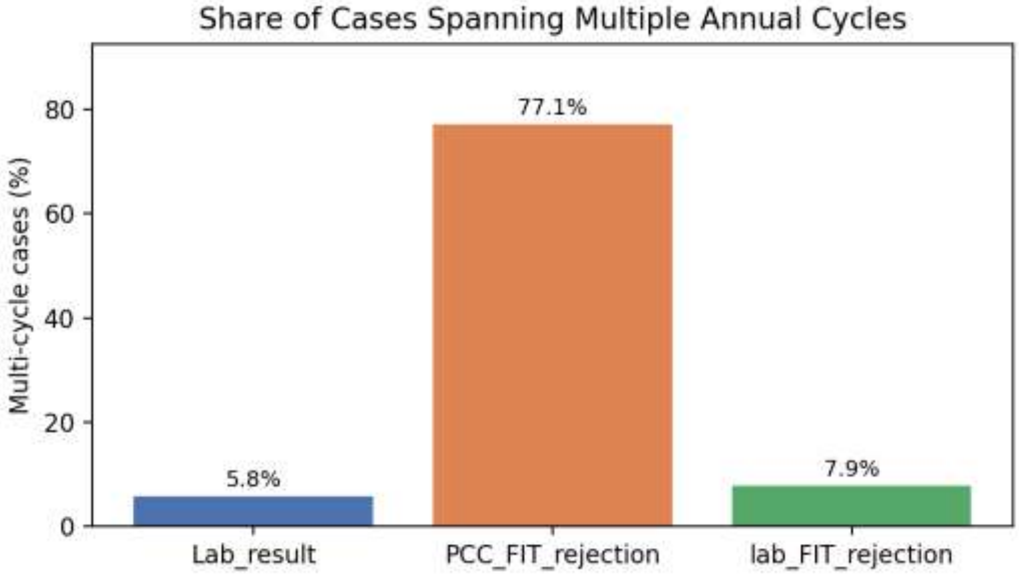

Figure S2. Multi-cycle participation rates. Higher multi-cycle proportions in PRE reflect longer observation windows and effective re-invitation protocols; POST maintains comparable engagement with shorter follow-up.

Together, these cycle metrics explain why large invitation/re-invitation loops correspond to ongoing engagement rather than process failure: repeated invitations keep participants in the program across multiple screening rounds.

S2. Cross-Period Conformance Overlay

We replayed POST (2024-2025) traces on the PRE (2022-2023) Inductive Miner model to localize drift. The POST-on-PRE overlay visualizes alignment diagnostics on the PRE model, highlighting elevated log moves at FIT_return and Invitation_mail, which aligns with the 31% dwell-time increase reported in Figure 5.

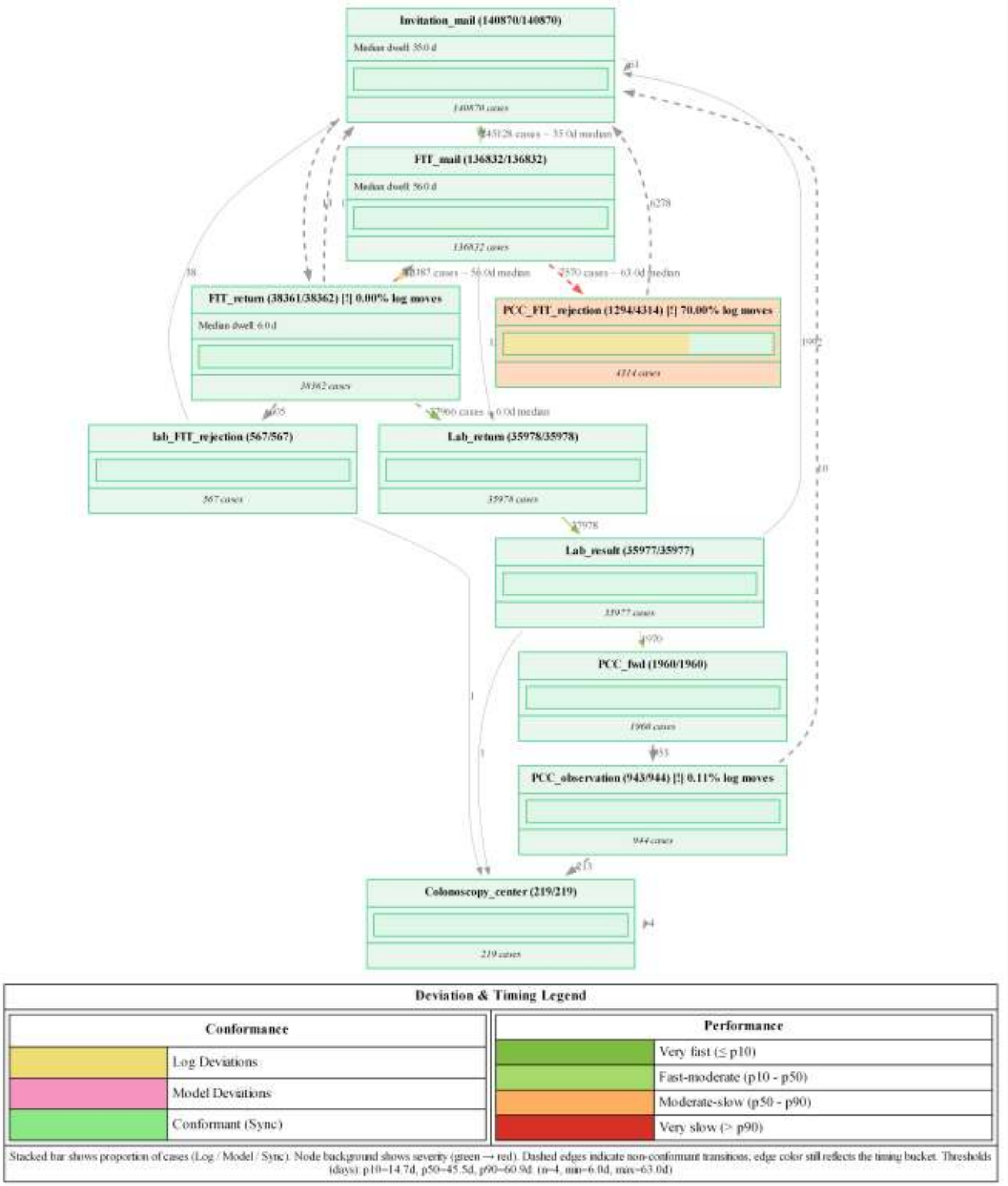

Figure S3. POST-on-PRE conformance overlay. The figure annotates the PRE model with POST log/model/sync moves, making visible the bottleneck concentrations that underpin the quantitative results.

S3. Activity-Level Conformance Analysis: Alignment-Based Assessment

Table S3: Alignment-Based Conformance Metrics (PRE vs. IDEAL)

Conformance checking via alignment decomposes trace-model discrepancies into three move types: log moves (activity in trace but not aligned with model-deviation), model moves (model expects activity which is not present in trace-missing step), and sync moves (perfect alignment). These metrics distinguish token replay (which tracks only visible activities) from alignment-based checking (which detects both extra and missing activities).

Table S3. Alignment-based conformance for PRE (discovered) and IDEAL (normative) models. Percentages show move distribution per activity occurrence. Activities in process flow order.

Activity	PRE Log%	PRE Model%	PRE Sync%	IDEAL Log%	IDEAL Model%	IDEAL Sync%
Invitation_mail	0.0%	0.0%	100.0%	0.0%	0.0%	100.0%
FIT_mail	0.0%	0.0%	100.0%	0.0%	2.9%	97.1%
FIT_return	0.0%	0.0%	100.0%	6.2%	0.0%	93.8%
Lab_return	0.0%	0.0%	100.0%	0.0%	0.0%	100.0%
Lab_result	0.0%	0.0%	100.0%	94.5%	0.0%	5.5%
PCC_fwd	0.0%	0.0%	100.0%	0.0%	0.1%	99.9%
PCC_observation	0.1%	0.0%	99.9%	0.1%	51.9%	48.0%
Colonoscopy_center	0.0%	0.0%	100.0%	0.2%	88.9%	10.9%
PCC_FIT_rejection	70.0%	0.0%	30.0%	100.0%	0.0%	0.0%
lab_FIT_rejection	0.0%	0.0%	100.0%	100.0%	0.0%	0.0%

Conformance Interpretation:

- PRE model: High sync rates (99.9-100.0% for most activities) indicate strong alignment with discovered behaviour. The main exception is PCC_FIT_rejection, with 70.0% log moves and 30.0% sync, showing cases that deviate from learned historical patterns.
- IDEAL model: Severe deviations concentrate in Lab_result, PCC_FIT_rejection, and lab_FIT_rejection. PCC_observation and Colonoscopy_center also show substantial model moves, meaning that the normative pathway expects steps that are frequently absent in practice.

- Lab_result: 94.5% log moves (the normative model does not expect lab results to appear in this form)

- PCC_FIT_rejection: 100.0% log moves (all rejection cases are unaligned in the IDEAL model)

- lab_FIT_rejection: 100.0% log moves (all lab rejection cases are unaligned in the IDEAL model)

- Model moves vs. Log moves: IDEAL shows model moves at FIT_mail (2.9%), PCC_observation (51.9%), and Colonoscopy_center (88.9%), indicating expected steps missing in real traces. PRE shows near-zero model moves because it was discovered from observed behaviour.
